# Identification and characterization of a novel orthoreovirus from American eels, *Anguilla rostrata*

**DOI:** 10.1128/spectrum.03607-25

**Published:** 2026-04-07

**Authors:** Jin-Xian Yang, Ying-Ying Li, Bin Sun, Qiang Chen, Jun-Qing Ge

**Affiliations:** 1 Institute of Biotechnology, Fujian Academy of Agricultural Sciences 107629, Fuzhou, China; University of Prince Edward Island, Charlottestown, Prince Edward Island, Canada

**Keywords:** hemorrhagic gill necrosis disease, *Anguilla rostrata*, Anguillid orthoreovirus, phylogenetic analysis

## Abstract

Hemorrhagic gill necrosis disease (HGND) has resulted in substantial economic losses in the farming of American eels (*Anguilla rostrata*). Although prior research implicates viruses like American eel adomavirus (AEAdoV) and Anguillid herpesvirus (AngHV), the primary etiological agent remains elusive. In this study, a novel virus was isolated from the HGND samples of American eel using eel ovary (EO) cell lines. Following isolation, the virus was subjected to comprehensive characterization encompassing morphological features, genomic organization, phylogenetic relationships, pathogenic potential, and epidemiological prevalence. The findings revealed that the virions are non-enveloped, icosahedral symmetry with a double-layered capsid and containing a genome composed of 10 double-stranded RNA segments, thereby confirming its classification within the genus *Orthoreovirus*. Phylogenetic analyses positioned this orthoreovirus in a distinct clade alongside largemouth bass reovirus (LMBRV) and piscine reovirus (PRV), occupying an intermediate position between the *Orthoreovirus* and *Aquareovirus* genera, indicating a unique evolutionary lineage within the *Spinareoviridae* family. This virus has been designated as Anguillid orthoreovirus (AORV). Experimental intraperitoneal challenge of American eels with AORV did not lead to apparent clinical manifestations. Epidemiological investigation revealed that AORV is an endemic orthoreovirus with an infection prevalence of 32.3% in farmed American eels. In summary, this study reports the successful isolation and comprehensive characterization of a novel orthoreovirus, AORV, from HGND samples of American eel. These findings provide a foundation for further investigations into the biology of AORV and offer novel insights toward future taxonomic refinement within the family *Spinareoviridae*.

**IMPORTANCE:**

A novel orthoreovirus, designated Anguillid orthoreovirus (AORV), was isolated from American eels with “hemorrhagic gill necrosis disease,” and forms a unique evolutionary lineage within the *Spinareoviridae* family. Although experimental challenges with AORV did not result in observable pathogenicity, epidemiological surveys indicated that approximately one-third of the farmed American eels displaying clinical signs of “hemorrhagic gill necrosis disease” tested positive for the virus, suggesting that AORV may be secondary to another primary pathogen or environmental stressor.

## INTRODUCTION

Members of the genus *Orthoreovirus* within the family *Spinareovirinae* (turreted) are characterized by a genome composing 10 double-stranded RNA (dsRNA) segments and infect a wide range of hosts, including primates, rodents, swine, birds, and teleost fish (1). Avian orthoreoviruses (ARV), for instance, can infect avian species and cause tenosynovitis, runting-stunting, and reoviral hepatitis, leading to high mortality and economic losses in the poultry industry (2, 3). Mammalian orthoreoviruses (MRV), isolated from bats, pigs, primates, and other hosts, are implicated in respiratory and gastrointestinal diseases, as well as encephalitis (4–6). However, reovirus-associated diseases in aquatic animals exhibit considerable taxonomic complexity, with pathogenicity varying not only among genera but also depending on viral strain and host specificity (7, 8). A well-defined species, *Orthoreovirus piscis*, encompasses numerous closely related isolates from salmonids and trout (9–12). For example, piscine orthoreovirus-1 (PRV-1) is associated with heart and skeletal muscle inflammation (HSMI) in Atlantic salmon (10, 13), while largemouth bass orthoreovirus (LMBRV) shows considerable genomic divergence from PRV (14). However, PRV remains refractory to conventional *in vitro* culture and must be propagated *in vivo* within salmon hosts (10, 15, 16), which hampers mechanistic studies of its virology and virus-host interactions. Although LMBRV has been successfully propagated in the bluegill fry (BF-2) cells (14), no subsequent studies have been reported to date. These findings emphasize the importance of cell lines in virological research and highlight the need for further study of orthoreoviruses in bony fish.

Conversely, viruses of the genus *Aquareovirus* possess a distinctive genome composed of 11 dsRNA segments and have been reported to infect fish and mollusks either asymptomatically or with pathogenic effects, including severe hemorrhagic mortality under certain circumstances (8). Representative strains include chum salmon aquareovirus (CSRV, Aquareovirus A) isolated from asymptomatic salmon (17), golden shiner reovirus (GSRV, Aquareovirus C) isolated from farmed grass carp and fathead minnows (18), *Scophthalmus maximus* reovirus (SMReV, Aquareovirus E) isolated from diseased turbot (19), American grass carp reovirus (AGCRV, Aquareovirus G) isolated from grass carp fingerlings (20), and marbled eel reovirus (MERV) isolated from *Anguilla marmorata* (21).

Eels (*Anguilla* spp.) are migratory freshwater fish that have gained prominence in the aquaculture industry in Southeast Asia. Since 2015, the American eel (*Anguilla rostrata*) has been widely introduced into aquaculture, facilitated by the relative abundance of glass eels and advances in standardized farming technologies. However, viral diseases have emerged as major threats to the health and welfare of farmed eels. Anguillid herpesvirus (AngHV) has been identified as the causative agent of “mucus sloughing and hemorrhagic septicemia disease” in American eel elvers (22). More recently, a novel epidemic disease characterized clinically by diffuse hemorrhaging and necrosis in gill filaments, accompanied by persistent mortality, has been observed in cultured American eels; this disease was named “hemorrhagic gill necrosis disease” (HGND) (23). The causative pathogen of the disease was suspected to be a viral agent, and American eel adomavirus (AEAdoV) was further isolated from clinical samples. However, no clinical signs or mortality were observed in eels experimentally inoculated with AEAdoV, indicating that the etiology of HGND remains unclear (23).

In this study, we successfully isolated a novel orthoreovirus, designated Anguillid orthoreovirus (AORV), from the diseased American eels with HGND. Phylogenetic analysis revealed that AORV occupies a distinct evolutionary position within the *Spinareovirinae* family. These findings provide valuable insights into the evolutionary relationships of reoviruses. Although experimental challenges with AORV alone did not result in observable pathogenicity, epidemiological surveys indicated a high prevalence of infection in samples suspected of HGND. The role of the AORV in the etiology of HGND warrants further investigation.

## MATERIALS AND METHODS

### Virus isolation

Eel ovary (EO) cells were cultured in Leibovitz’s L-15 medium (HyClone, Logan, UT, USA) supplemented with 10% fetal bovine serum (FBS) (Gibco, Carlsbad, CA, USA), 100 U/mL penicillin, and 100 U/mL streptomycin at 27°C. In October 2020, spleen and kidney tissues were aseptically collected from moribund American eels exhibiting clinical signs of HGND at a farm in Fujian Province, China. Tissue samples from four individual eels were pooled for virus isolation. The tissues were homogenized in phosphate-buffered saline (PBS) at a 1:10 (wt/vol) ratio and centrifuged at 10,000 × *g* for 10 min. The resulting supernatants were filtered through a 0.45 μm syringe filter and inoculated onto monolayers of EO cells maintained in L-15 medium supplemented with 2% FBS at 27°C. Cells were observed daily using an inverted light microscope (Nikon TE-2000). At 7 days post-inoculation (dpi), the cells were subcultured for subsequent passages. A consistent cytopathic effect (CPE) was observed from the third passage onward. Cell culture supernatants were collected for further analysis.

### Virion purification and electron microscopy

To purify virions, EO cells were inoculated with the collected cell culture supernatants. When the majority of cells exhibited CPE, both the cells and the supernatants were harvested and centrifuged at 10,000 × *g* for 60 min. The supernatants were then ultra-centrifuged at 160,000 × *g* for 2 h to pellet the virions. The resulting pellet was re-suspended in sterile PBS and carefully layered onto a discontinuous sucrose gradient (60%, 50%, 40%, 30%, and 20% sucrose in PBS), followed by ultra-centrifugation at 160,000 × *g* for 2 h. Then the interlayer bands were collected, diluted with PBS, and centrifuged again at 160,000 × *g* for 1 h to remove residual sucrose. Finally, the viral pellets were re-suspended in PBS and stored temporarily at 4°C. The purified virions were placed on a copper mesh, dried, and negatively stained with phosphotungstic acid and examined using a Hitachi HT7700 transmission electron microscope.

### Viral titer determination

Viral titers were determined using supernatants from virus-infected EO cells. EO cells were seeded into 96-well cell culture plates at a density of 10^4^ cells/0.1 mL in each well. Ten-fold serial dilutions of the virus, prepared in L-15 medium supplemented with 2% FBS, were added to the wells, and the plates were incubated at 27°C. The presence of CPE in each well was monitored and recorded at 6 dpi. The 50% tissue culture infective dose (TCID_50_) was calculated using the Reed-Muench method.

### 5-Iodo-2′-deoxyuridine assay

5-Iodo-2′-deoxyuridine (IDU), a thymidine analog that is incorporated into DNA, was used to identify DNA viruses. EO cells were infected with the virus. At 3 h post-infection (hpi), cells were washed and cultured in medium containing 50 μg/mL IDU. At 120 hpi, supernatants were collected, and viral titers were determined via TCID_50_ assay as described above. Each assay was performed in triplicate, with virus-infected cultures without IDU treatment serving as controls.

### Viral genomic RNA electrophoresis

Total RNA was extracted from 0.1 mL of purified virions using TRIzol reagent (Invitrogen, ThermoFisher Scientific, USA), and RNA quality was assessed using a NanoDrop ND-1000 UV-Vis spectrophotometer (NanoDrop Technologies, USA). For polyacrylamide gel electrophoresis (PAGE) analysis, 500 ng of RNA was mixed with 6× RNA loading buffer, loaded onto an 8% vertical slab PAGE gel, and electrophoresed in 4% formaldehyde-MOPS buffer at a constant current of 30 mA for 20 h. The viral genome electropherotype was visualized using a silver staining kit (Sangon Biotech, Shanghai, China) according to the manufacturer’s instructions.

### Viral genome sequencing, assembly, and annotation

Total RNA extracted from purified virions was subjected to next-generation sequencing (NGS) by Tsingke Biotechnology Co., Ltd. (Beijing, China). Briefly, Illumina NovaSeq 6000 PE150 sequencing technology was utilized for deep sequencing. Raw reads were quality-trimmed using Trimmomatic and filtered to obtain high-quality sequences (24). D*e novo* assembly was performed using metaQUAST, with contigs manually aligned and reassembled (25). Viral-specific primers, designed based on NGS data, were used to amplify the full-length genome using the HiScript 1st Strand cDNA Synthesis Kit, 2×Phanta Max Master Mix, and SMARTer RACE 5′/3′ Kit (all from Vazyme Biotech, Nanjing, China). Sanger sequencing was performed to fill gaps, and the complete genome was assembled using SeqMan software (Lasergene, DNASTAR, ver. 7.1.0) via overlapping sequence alignment. Homologous sequences were identified using BLAST at the National Center for Biotechnology Information server (NCBI). Based on nucleotide sequence identities, the 10 segments of the viral genome were named and deposited in GenBank (1, 26). The isolated virus was designated Anguillid orthoreovirus (AORV).

### Phylogenetic analysis

The AORV L3 segment, encoding the RNA-dependent RNA polymerase (RdRp), was selected for phylogenetic analysis due to its high conservation among viral proteins in the order *Reovirales* (1, 8). Nucleotide sequences of the AORV L3 segment and corresponding RdRp segments from GenBank (listed in Table S1) were analyzed. To further investigate evolutionary relationships within the *Orthoreovirus* and *Aquareovirus* genera, phylogenetic analyses were also conducted using amino acid sequences of the outer clamp protein (σ3/B) and the homologous VP7 protein of aquareovirus (listed in Table S2). Multiple sequence alignments were performed using the Clustal W program of the MEGA software (ver. 11.0.13), and phylogenetic trees were constructed via the Neighbor-Joining method with 1,000 bootstrap replicates to assess branch reliability.

### Real-time quantitative PCR assay

A set of specific primers and a corresponding labeled probe targeting a conserved region (940–1,056 nt) of the L3 segment were designed for the detection of AORV. Extracted RNA samples were mixed with the One Step PrimeScript III real-time quantitative PCR (RT-qPCR) Mix (TaKaRa, Beijing, China). The reaction protocol included a reverse transcription step at 52°C for 5 min followed by 95°C for 10 s. Amplification was then carried out over 40 cycles, each consisting of denaturation at 95°C for 5 s and annealing/extension at 60°C for 30 s. For quantification purposes, a 564 bp fragment (813–1,376 nt) of the L3 segment was cloned into the pCE2-TA plasmid vector (Vazyme Biotech, Nanjing, China) to generate a standard curve.

### Viral pathogenicity assay

Sixty healthy American eels weighing 150–200 g were randomly divided into control and experimental groups. Eels in the experimental group were intraperitoneally injected with 1.0 mL of cell supernatant containing 10^6^ TCID_50_/mL of AORV, while controls were injected with 1.0 mL of L-15 medium. Eels were maintained in aquaria under standard culture conditions at 26°C ± 1°C and monitored daily for clinical signs, behavioral changes, and mortality over 50 days. At 28 dpi, three eels from the challenged group were euthanized, and tissues, including blood, heart, liver, spleen, kidney, and gill, were collected for virus re-isolation and viral load quantification via RT-qPCR. Control group eels underwent identical sampling procedures.

### Epidemiological investigation

Based on preliminary clinical and pathological examinations, 31 cases of farmed American eels suspected of HGND, characterized by diffuse hemorrhage and necrosis in the gill filaments, were identified. Tissue samples from the diseased eels were collected, and total RNA was extracted using TRIzol reagent (Thermo Fisher Scientific, USA). The presence of AORV was evaluated via the specific RT-qPCR assay, and the viral infection prevalence was calculated.

## RESULTS

### Virus isolation and identification

**Fig 1:**
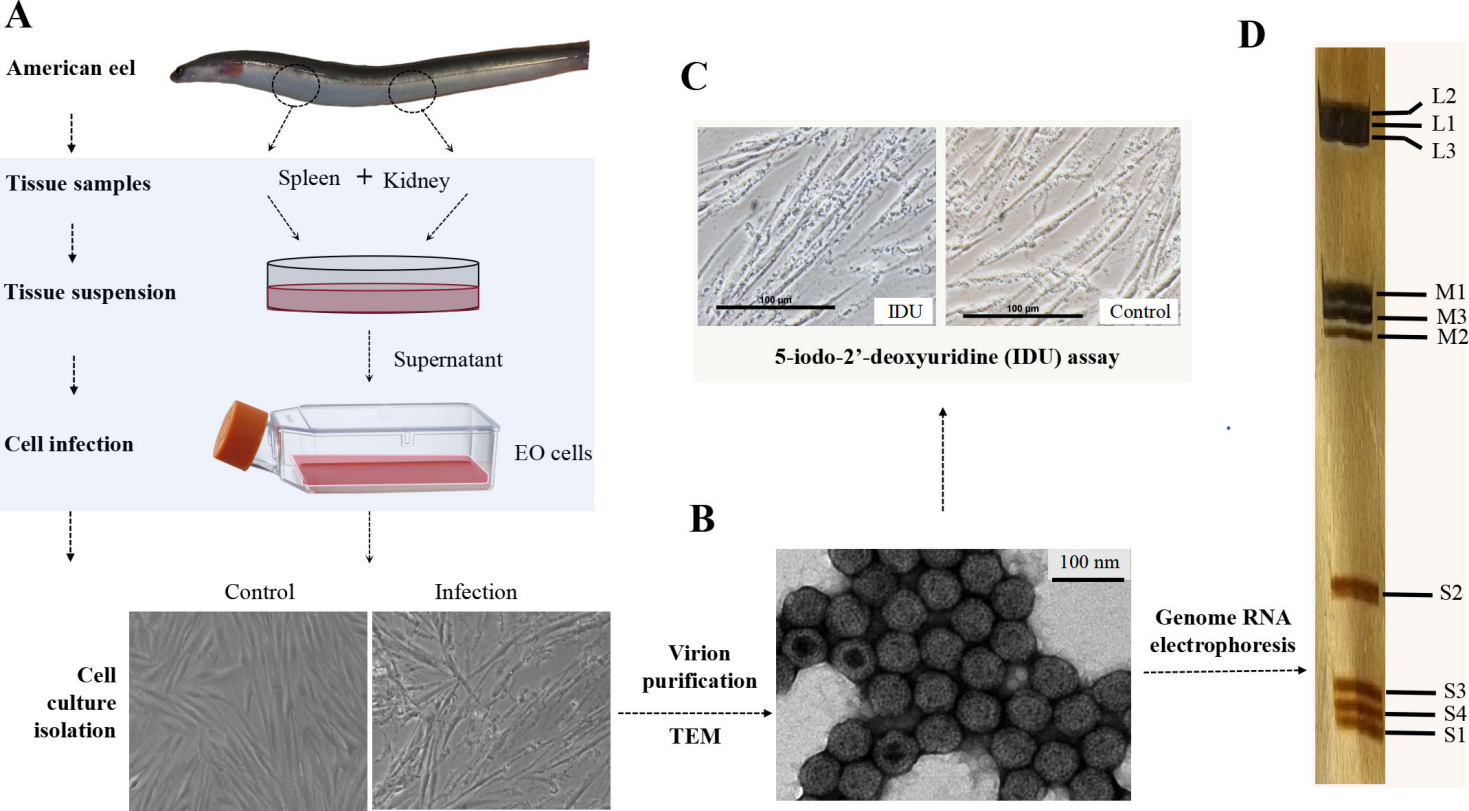
The isolation and identification of a novel orthoreovirus from American eels. (**A**) A viral strain was isolated with distinctive cytopathic effect in EO cells. (**B**) The morphology of the virion was observed through negative staining and electron microscopy. (**C**) The isolated virus displayed resistance to the DNA synthesis inhibitor IDU. (**D**) The electrophoretic profile of the viral genomic RNA.

Spleen and kidney tissue homogenates derived from American eels exhibiting HGND were inoculated into EO cell cultures for serial passaging. By the third passage, cytopathic effects (CPE) were observed at 3 dpi, characterized by cytoplasmic degradation, the formation of slender and opaque residual cytoskeletal structures, and localized cellular disintegration into small fragments (Fig. 1A). These pathological changes progressed to involve the entire EO cell monolayer by 5 dpi. The CPE remained stable and consistent from the 3rd to 10th passage, indicating successful viral isolation and propagation in EO cells. Subsequently, virions were purified from the supernatant of infected EO cultures using sucrose gradient ultracentrifugation. Transmission electron microscopy showed that the purified virions were non-enveloped, exhibited icosahedral symmetry with a double-layered capsid, measured 75–85 nm in diameter, with some containing electron-dense cores (Fig. 1B). Besides, the isolated virus was resistant to the DNA synthesis inhibitor IDU, showing no significant difference in CPE and viral titers between IDU-treated and control groups (Fig. 1C), suggesting it is an RNA virus. Polyacrylamide gel electrophoresis (PAGE) analysis further confirmed that the virus comprises a distinct segmented RNA genome (Fig. 1D). Collectively, these results identify the isolated virus as a non-enveloped RNA virus with a segmented genome.

### Virus sequencing, structural characteristics, and phylogenetic analysis

**Fig 2:**
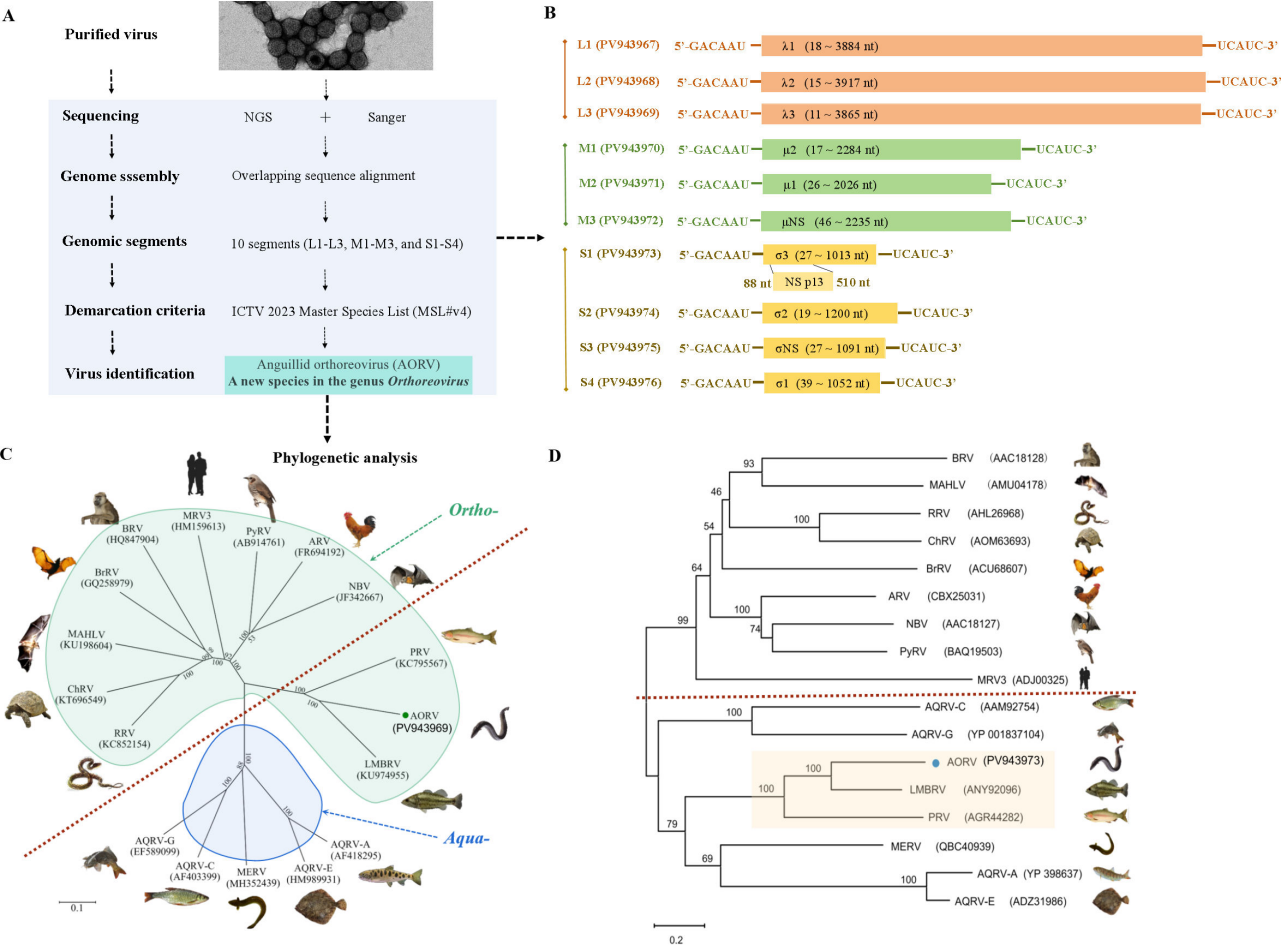
The AORV genome sequencing and the phylogenetic analysis. (**A**) Outline of the genome sequencing procedure. (**B**) The schematic diagram of the AORV genome segments. (**C**) Phylogenetic relationships between AORV and the selected reoviruses based on nucleotide sequences of the RNA-dependent RNA polymerase (RdRp). (**D**) Phylogenetic relationships were established using amino acid sequences of the outer clamp protein of AORV and the selected reoviruses. The RdRp and the outer clamp sequences, along with their corresponding GenBank accession numbers, are listed in Tables S1 and S2, respectively. Sequence alignments were conducted using the Clustal W algorithm, and phylogenetic trees were constructed using the Neighbor-Joining method of the MEGA software, with branch evaluation through 1,000 bootstrap replicates.

Utilizing the NGS data complemented by Sanger sequencing, the complete viral genome was assembled through overlapping sequence alignment (Fig. 2A). The genome comprises 10 double-stranded RNA segments ranging from 1,080 to 3,965 nt in length (Table 1; Fig. 2B), classified into three size-based categories: large (L1-3), medium (M1-3), and small (S1-4) groups. Each segment of the viral genome encodes a single protein, including a core spike protein, confirming its taxonomic placement within the genus *Orthoreovirus*. Therefore, the virus was designated as AORV, with detailed annotations summarized in Table 1. Additionally, AORV possesses a conserved 5′-terminal sequence (GACAAU) on the positive strand of each genomic segment, while the 3′-terminal motif (UCAUC) is conserved across all reovirus strains (Table 2; Fig. 2B). The lengths of the 5′- and 3′- non-translated regions (NTRs) vary among segments, with the longest 5′-NTR observed in M3 (10–45 nt) and the longest 3′-NTR in S2 (45–135 nt) (Table 1). Notably, the non-structural p13 protein, encoded by ORF2 within the S1 segment, is predicted to be a transmembrane protein with its N-terminal region containing a putative transmembrane domain (TMD: A35VIVLAALLVISLTWAIITAV56). These genomic features distinguish AORV from other orthoreoviruses and aquareoviruses.

**TABLE 1:** AORV genome coding assignments and predicted protein characteristics^*a*^

Genome segment	Nucleotide sequence				Putative coding protein					Predicted function
	Molecular size (nt)	5′-NTRs (nt)	3′-NTRs (nt)	ORF	Protein nomenclature	Virion location	Amino acid (aa)	Predicted mass (kDa)	*P* value	
L1	3,932	17	48	18–3,884	λ1	Core shell	1,288	141.83	5.87	Major inner capsid protein, helicase, RNA triphosphatase
L2	3,965	14	48	15–3,917	λ2	Core turret	1,300	145.15	5.15	Core spike, guanynyl transferase, methyl transferase
L3	3,910	10	45	11–3,865	λ3	Core RdRp	1,284	143.96	8.70	Minor inner capsid protein, RNA-dependent RNA polymerase
M1	2,354	16	70	2,284	μ2	Core NTPase	755	85.80	8.31	Minor inner capsid protein, nucleotide triphosphatase phosphohydrolase
M2	2,137	25	111	26–2,026	μ1	Outer shell	666	71.84	6.37	Outer capsid protein, membrane penetration, apoptosis
M3	2,316	45	81	46–2,235	μNS	NS protein	729	80.91	4.83	Non-structural protein
S1	1,080	26	67	27–1,013	σ3	Outer clamp	328	35.87	8.67	Major outer capsid protein, dsRNA binding protein, zinc metalloprotein, modulation of cellular interferon, reovirus viral attachment protein
				88–510	p13	NS protein	140	14.48	5.56	Non-structural protein, cytolytic but not fusogenic integral membrane protein
S2	1,335	18	135	19–1,200	σ2	Core clamp	393	44.07	6.4	Major inner capsid protein, RNA binding
S3	1,137	26	46	27–1,091	σNS	NS protein	354	38.91	8.27	Non-structural protein, involved in virus inclusion formation
S4	1,128	38	76	39–1,052	σ1	Outer fiber	337	37.26	5.53	Outer capsid protein (virus attachment), cell tropism, pathways of viral spread *in vivo*, virulence

^*a*^ The genome segments L1–S4 were deposited in the GenBank with the accession numbers PV943967–PV943976, respectively.

**TABLE 2:** Conserved terminal NTRs among the *Orthoreovirus* and *Aquariovirus*

Virus genus	General virus species	5′-End	3′-End
*Mammalian orthoreovirus*	Mammalian orthoreovirus (MRV 3)	5′-GCUA	UCAUC-3′
*Avian orthoreovirus*	Avian orthoreovirus (ARV)	5′-GCUUUUU	
*Nelson Bay orthoreovirus*	Nelson Bay orthoreovirus (NBV)	5′-GCUUUA	
*Baboon orthoreovirus*	Baboon orthoreovirus (BRV)	5′-GUAAAUUU	
*Reptilian orthoreovirus*	Reptilian orthoreovirus (RRV)	5′-GUU(^A^/_C_)UUUU	
*Mahlapitsi orthoreovirus*	Mahlapitsi orthoreovirus (MAHLV)	5′-GGUCA	
*Broome orthoreovirus*	Broome orthoreovirus (BrRV)	5′-GUCAA	
*Neoavian orthoreovirus*	Pycnonotidae orthoreovirus (PyRV)	5′-GCCUUUC	
*Testudine orthoreovirus*	Chelonian orthoreovirus (ChRV)	5′-GUU(^A^/_C_)UUC	
Tentative *Orthoreovirus*	Anguillid orthoreovirus (AORV)	5′-GACAAU	
*Piscine orthoreovirus*	Piscine orthoreovirus (PRV-1)	5′-GAUAA^A^/_U_	
Tentative *Orthoreovirus*	Largemouth bass reovirus (LMBRV)	5′-GACAU^*a*^	
*Aquareovirus A*	Chum salmon reovirus (CSRV)	5′-GUUUUA^U^/_G_	
*Aquareovirus C*	Golden shiner reovirus (GSRV)	5′-GUUAUA^U^/_G_	
*Aquareovirus E*	Scophthalmus maximus reovirus (SMReV)	5′-GUUUUA^U^/_G_	
*Aquareovirus G*	American grass carp reovirus (AGCRV)	5′-GUUUUA^U^/_A_	
Tentative *Aquareovirus*	Marbled eel reovirus (MERV)	–	–

^*a*^ NTRs of the LMBRV based on the genome segment L3 sequencing (14).

Phylogenetic analyses based on RdRp nucleotide sequences revealed that AORV, along with the fish-derived LMBRV and PRV, form a distinct evolutionary clade located between the genera *Orthoreovirus* and *Aquareovirus* (Fig. 2C). Moreover, analysis of the outer clamp protein indicated that these three viruses exhibit closer phylogenetic affinity to aquareoviruses than to orthoreoviruses, suggesting the existence of a unique lineage bridging the two genera and narrowing the taxonomic boundary between the genus *Orthoreovirus* and *Aquareovirus* (Fig. 2D).

### Viral pathogenicity

During the 50-day trial, American eels intraperitoneally inoculated with AORV did not exhibit clinical signs. However, RT-qPCR analysis confirmed the presence of AORV in tissues at 28 dpi (data not shown), and the virus was successfully re-isolated from the spleen, kidney, and gill tissues. Inoculation of the re-isolated virus onto EO cell monolayers induced typical and persistent CPEs (data not shown). These results indicate that AORV can establish systemic infection in American eels without observable pathogenicity; however, whether the AORV is a benign or an endemic co-infection virus requires further elucidation.

### Epidemiological investigation of viral infections

An epidemiological investigation was conducted on 31 farmed American eels exhibiting typical clinical signs of HGND, including diffuse hemorrhage and necrosis within the gill filaments. These specimens were analyzed via RT-qPCR and demonstrated a prevalence rate of 32.3% (10/31) for AORV infection (Table 3). These findings indicated that AORV is an endemic orthoreovirus affecting farmed American eels; however, its precise pathogenic role remains to be elucidated.

**TABLE 3:** The prevalence of AORV infection in the collected eel tissue samples using RT-qPCR analysis

Serial no.	Sample number	Clinical sign^*a*^	Tissue for detection	AORV detection
1	2019812	Typical	Gill	Negative
2	2017314-2	Typical	Liver, spleen, kidney, gill	Negative
3	171129	Typical	Gill	Negative
4	1768^*c*^	Typical	Gill	Positive
5	23116	Typical	Gill	Negative
6	201025^*b*^	Typical	Liver, spleen, kidney, gill	Positive
7	171117^*c*^	Typical	Gill	Positive
8	2018118	Typical	Gill	Negative
9	20911	Typical	Gill	Negative
10	22127	Typical	Liver, spleen, kidney	Negative
11	23818	Typical	Gill	Negative
12	2365	Typical	Gill	Negative
13	231019	Typical	Liver, spleen, kidney, gill	Negative
14	24611	Typical	Gill	Negative
15	1781	Typical	Gill	Positive
16	23731	Typical	Gill	Negative
17	20821	Typical	Liver, spleen, kidney	Positive
18	2458	Typical	Liver, spleen, kidney, gill	Negative
19	191014	Typical	Gill	Negative
20	1987	Typical	Liver, spleen, kidney	Negative
21	2019923	Typical	Gill	Negative
22	XSD201854^*c*^	Typical	Liver, spleen, kidney	Positive
23	XSD180522	Typical	Liver, spleen, kidney, gill	Positive
24	2020330	Typical	Liver, spleen, kidney	Negative
25	2019821	Typical	Gill	Negative
26	17815	Typical	Liver, spleen, kidney	Negative
27	20171227	Typical	Gill	Positive
28	2019821	Typical	Liver, spleen, kidney	Negative
29	19923	Typical	Liver, spleen, kidney	Negative
30	202019	Typical	Liver, spleen, kidney	Positive
31	21414	Typical	Liver, spleen, kidney	Positive

^*a*^ Diagnosis is established based on the pathological changes characterized by diffuse hemorrhage and necrosis within the gill filaments.

^*b*^ The AROV strain is reported in this manuscript.

^*c*^ The homologous AROV strain was successfully isolated.

## DISCUSSION

HGND has emerged as a serious threat to the health and welfare of farmed American eels, resulting in considerable economic losses. Although AEAdoV was previously isolated from eels exhibiting HGND signs, it was determined to be non-pathogenic, leaving the precise etiology of HGND unclear and necessitating further investigation (23). Notably, the hemorrhagic signs and pathological changes of HGND in American eels closely resemble those of viral endothelial cell necrosis of eel (VECNE) in Japanese eels (*Anguilla japonica*), which is associated with Japanese eel endothelial cell-infecting virus (JEECV) (27), a homolog of the AEAdoV. Furthermore, marbled eels (*Anguilla marmorata*) exhibiting petechial skin hemorrhages were found to be infected with both marbled eel reovirus (MERV, a tentative aquareovirus) and marbled eel adomavirus (MEAdoV), although their etiological roles remain unclear (21, 28, 29). In this study, a novel reovirus, designated AORV, was isolated from diseased American eels and taxonomically identified as a new species within the genus *Orthoreovirus*. Epidemiological analyses revealed that AORV is an endemic orthoreovirus with an infection prevalence of 32.3% in typical HGND samples obtained from American eels. However, intraperitoneal challenge of American eels with AORV did not lead to apparent clinical manifestations. These findings suggest that AORV alone may be insufficient to cause overt disease or that it may represent a benign viral agent. Similarly, many aquatic viruses are now recognized as components of the normal fish virome that may become detectable during periods of stress or immunosuppression caused by other primary pathogens. This phenomenon underscores the importance of distinguishing between viral presence and viral pathogenicity in disease investigations. Therefore, it is imperative to conduct epidemiological studies comparing HGND eels with healthy eels. Furthermore, the potential roles of both AEAdoV and AORV in the pathogenesis of HGND warrant further elucidation through comprehensive investigation.

*In vitro* infection of EO cells with AORV induced cytopathic effects characterized by cytoplasmic loss without syncytium formation. This non-fusogenic phenotype is consistent with that of PRV, which causes heart and skeletal muscle inflammation (HSMI) in Atlantic salmon (*Salmo salar*) and expresses a non-fusogenic NS p13 protein that induces cytotoxicity without cell fusion (10, 30). The PRV NS p13 protein was also predicted to contain conserved TMDs (Y26VLNAGIGLVCLIMLSLLWSLI47), which are analogous to those found in the NS p13 protein of AORV. Notably, despite this structural similarity, the two share only 30.9% amino acid identity as determined by multiple sequence alignment (31). Given that PRV NS p13 has been experimentally confirmed as a membrane-localized, cytolytic, and non-syncytogenic protein (non-FAST protein) (30), it is proposed that AORV is also a non-fusogenic orthoreovirus. This is consistent with the observed CPE in AORV-infected EO cells. In contrast, aquareoviruses typically induce syncytium formation via fusion-associated small transmembrane (FAST) proteins (32–35), highlighting a fundamental phenotypic distinction between AORV and PRV compared to the isolates of the aquareoviruses.

PRV was initially identified in Atlantic salmon affected by HSMI and proposed as a new species, *Orthoreovirus piscis*, within the genus *Orthoreovirus* (1, 26). Although certain PRV strains exhibit high infectivity with low virulence, the virus generally establishes systemic infections with high viral loads by replicating predominantly within erythrocytes (36–38). Over the past decade, substantial research has elucidated various aspects of PRV pathogenesis, taxonomy, epidemiology, and biology (10, 15, 26, 39). However, these advances have largely relied on molecular diagnostics and *ex vivo* models using salmon erythrocytes, as PRV remains refractory to propagation in conventional cell culture systems (8, 37, 38). This limitation has hindered detailed mechanistic studies of viral replication and host interactions. The successful isolation and propagation of AORV in EO cells thus provides a valuable *in vitro* model for investigating the pathogenic mechanisms and evolutionary ecology of finfish orthoreoviruses.

According to the International Committee on Taxonomy of Viruses (ICTV) (MSL#39.v4), the genus *Orthoreovirus* comprises 10 species and is delineated based on established demarcation criteria. These criteria emphasize sequence identity among homologous genome segments: conserved core proteins typically exhibit >85% amino acid identity within species and <65% between species, whereas more variable outer capsid proteins generally show >55% identity within species and <35% between species (1). In the case of AORV, the core RNA-dependent RNA polymerase (RdRp) shares 73.4% and 64.3% amino acid identity with LMBRV and PRV, respectively, while the major outer capsid protein (outer clamp) showed notably lower identities of 52.0% and 32.3%, respectively (Table S2). Based on these taxonomic criteria and distinctive viral features, AORV is proposed to be a novel species of the genus *Orthoreovirus*.

The classification of *Aquareovirus* and *Orthoreovirus* into two distinct genera is well established and reflects fundamental differences in their biological and molecular characteristics (1, 8). Phylogenetic analysis based on RdRp nucleotide sequences revealed that AORV, along with LMBRV and PRV, forms an independent clade situated between *Orthoreovirus* and *Aquareovirus*, suggesting a unique evolutionary position within the *Spinareovirinae* family. Further multiple sequence alignment of the outer-clamp protein amino acid sequences indicated that this clade clusters more closely with aquareoviruses, implying that AORV represents a distinct lineage bridging the two genera and narrowing the taxonomic distinction between *Orthoreovirus* and *Aquareovirus*. These findings provide novel insights that may inform future taxonomic refinement within the family *Spinareoviridae* (turreted reoviruses).

### Conclusion

In this study, we successfully isolated and characterized a novel orthoreovirus, designated Anguillid orthoreovirus (AORV), from HGND samples of American eels (*Anguilla rostrata*). AORV propagates efficiently in EO cell lines, inducing distinct CPEs. Morphological and genomic analyses identified AORV as a non-enveloped, icosahedral virus with a double-layered capsid and a genome comprising 10 double-stranded RNA segments, thereby confirming its classification within the genus *Orthoreovirus*. Phylogenetic analyses positioned AORV in close relation to LMBRV and PRV, collectively forming a unique evolutionary clade referred to as the finfish orthoreoviruses and situated intermediate to the genera *Orthoreovirus* and *Aquareovirus*, underscoring its distinctive phylogenetic status. Epidemiological surveys revealed that AORV is endemic among farmed American eels, with an infection prevalence of 32.3%. Notably, experimental infections with AORV alone were insufficient to induce overt disease, implying that AORV is a benign virus. These findings establish a foundational basis for further research into the biology of AORV and contribute novel insights pertinent to the taxonomic refinement within the family *Spinareoviridae*.

## Data Availability

The complete genomic sequences of 10 segments have been deposited in GenBank under accession numbers PV943967–PV943976.

## SUPPLEMENTAL MATERIAL

Supplemental material spectrum.03607-25-s0001.docx Tables S1 and S2.

ASM does not own the copyrights to Supplemental Material that may be linked to, or accessed through, an article. The authors have granted ASM a non-exclusive, world-wide license to publish the Supplemental Material files. Please contact the corresponding author directly for reuse.
