# Supplementary material for: Identification and characterization of a novel orthoreovirus from American eels, Anguilla rostrata
Source: Microbiol Spectr. 2026 Apr 7;14(5):e03607-25. doi: 10.1128/spectrum.03607-25 (PMC13141839; doi:10.1128/spectrum.03607-25)
Supplement: Supplemental material — Tables S1 and S2. [file spectrum.03607-25-s0001.docx]

**Supplementary Materials**

Table S1 The RNA-dependent RNA polymerase (RdRp) segments and the λ3/B and VP2 homologs utilized in the comparative phylogenetic analysis

| Virus name | Virus strains | GenBank accession no. | Genome segment locus | Nucleotide identities  ( %)* |
| --- | --- | --- | --- | --- |
| AORV | Anguillid orthoreovirus, 1025 | PV943969 | L3 / λ3 |  |
| LMBRV | Largemouth bass reovirus, Pine Lake | KU974955 | L3 / λ3 | 66.4 |
| PRV | Piscine orthoreovirus, CGA280-05 | KC795567 | L3 / λ3 | 61.5 |
| BrRV | Broome reovirus | GQ258979 | L3 / λ3 | 49.6 |
| ARV | Avian orthoreovirus, AVS-B | FR694192 | L2 / λB | 47.8 |
| MRV3 | Mammalian orthoreovirus 3, T3D | HM159613 | L1 / λ3 | 51.3 |
| NBV | Nelson Bay orthoreovirus, Pulau | JF342667 | L2 / λB | 52.9 |
| BRV | Baboon orthoreovirus, USA/11-1993 | HQ847904 | L2 / λB | 48.2 |
| MAHLV | Mahlapitsi orthoreovirus, 2511 | KU198604 | L3 / λB | 49.5 |
| PyRV | Pycnonotidae orthoreovirus, Pycno-1 | AB914761 | L2 / λB | 49.7 |
| RRV | Reptilian orthoreovirus, 47/02 | KC852154 | L3 / λB | 48.9 |
| ChRV | chelonian orthoreovirus, CH1197/96 | KT696549 | L3 / λB | 48.6 |
| AQRV-A | Chum salmon reovirus, CSRV | AF418295 | Seg. 2 /VP2 | 46.5 |
| AQRV-C | Golden shiner reovirus, GSRV | AF403399 | Seg. 2 /VP2 | 45.9 |
| AQRV-E | Scophthalmus maximus reovirus, SMReV | HM989931 | Seg. 2 /VP2 | 51.1 |
| AQRV-G | American grass carp reovirus, AGCRV_PB01-155 | EF589099 | Seg. 2 /VP2 | 45.6 |
| MERV | Marbled eel reovirus, MERV-1 | MH352439 | Seg. 2 /VP2 | 46.9 |

*the percentage identities of the AORV RdRp nucleotide sequence compared with selected reoviruses were determined by alignment using the Clustal W algorithm.

Table S2 The amino acid sequences of the outer clamp proteins (σ3 / σB and the homologous VP2) utilized for the comparative sequence analysis and the phylogenetic analysis

| Virus name | Virus isolate | GenBank accession no. | Genome segment locus | Amino acid identities  ( %)* |
| --- | --- | --- | --- | --- |
| AORV | Anguillid orthoreovirus, 1025 | PV943973 | S1 /σ3 |  |
| LMBRV | Largemouth bass reovirus, Pine Lake | ANY92096 | S1 /σ3 | 52.0 |
| PRV | Piscine orthoreovirus, CGA280-05 | AGR44282 | S1 /σ3 | 32.3 |
| BrRV | Broome reovirus | ACU68607 | S2 /σB | 9.3 |
| ARV | Avian orthoreovirus, AVS-B | CBX25031 | S3 /σB | 13.0 |
| MRV3 | Mammalian orthoreovirus 3, T3D | ADJ00325 | S4 /σB | 9.7 |
| NBV | Nelson Bay orthoreovirus, Pulau | AAR13236 | S4 /σB | 12.5 |
| BRV | Baboon orthoreovirus, USA/11-1993 | AAC18128 | S2 /σB | 8.9 |
| MAHLV | Mahlapitsi orthoreovirus, 2511 | AMU04178 | S2 /σB | 11.1 |
| PyRV | Pycnonotidae orthoreovirus, Pycno-1 | BAQ19503 | S3 /σB | 13.2 |
| RRV | Reptilian orthoreovirus, 47/02 | AHL26968 | S3 /σB | 9.5 |
| ChRV | chelonian orthoreovirus, CH1197/96 | AOM63693 | S3 /σB | 13.2 |
| AQRV-A | Chum salmon reovirus, CSRV | YP398637 | Seg. 10 / VP7 | 9.3 |
| AQRV-C | Golden shiner reovirus, GSRV | AAM92754 | Seg. 10 / VP7 | 11.8 |
| AQRV-E | Scophthalmus maximus reovirus, SMReV | ADZ31986 | Seg. 10 / VP7 | 10.7 |
| AQRV-G | American grass carp reovirus, AGCRV_PB01-155 | YP001837104 | Seg. 10 / VP7 | 9.3 |
| MERV | Marbled eel reovirus, MERV-1 | QBC40939 | Seg. 10 / VP7 | 7.8 |

*the percentage identities of the amino acid sequences of the AORV outer clamp proteins (σ3 / σB and the homologous VP2) compared with selected reoviruses were determined by alignment using the Clustal W algorithm.
